# Placental mesenchymal dysplasia complicated with sudden fetal demise and amniotic fluid embolism: a case report

**DOI:** 10.1186/s12884-022-05261-2

**Published:** 2022-12-09

**Authors:** Shao-Jing Wang, Li-Ling Lin, Wei-Chih Chen

**Affiliations:** Department of Gynecology and Obstetrics, Taichung Veterans General Hospital, Taiwan Taichung,

**Keywords:** Amniotic fluid embolism, Fetal growth restriction, Intrauterine fetal demise, Placental mesenchymal dysplasia, Placenta previa, Preeclampsia

## Abstract

**Background:**

Placenta mesenchymal dysplasia (PMD) is a rare placental anomaly associated with various fetal and maternal complications. Whether close ultrasound surveillance can prevent intrauterine fetal demise (IUFD) in patients with PMD is still under investigation. Amniotic fluid embolism (AFE) is a rare, lethal, and unpredictable maternal complication that has never been described in association with PMD. Here, we report a case of PMD, in which the fetus eventually demised in utero despite weekly color Doppler monitoring, and the mother subsequently encountered AFE during delivery.

**Case presentation:**

A 43-year-old woman who had received three frozen embryo transfer, was found to have a singleton pregnancy with an enlarged multi-cystic placenta at 8 weeks’ gestation. Fetal growth restriction (FGR) was noted since the 21^st^week. The fetus eventually demised in-utero at 25 weeks despite weekly color Doppler surveillance. Cesarean section was performed under general anesthesia due to placenta previa totalis and antepartum hemorrhage. During surgery, the patient experienced a sudden blood pressure drop and desaturation followed by profound coagulopathy. AFE was suspected. After administration of inotropic agents and massive blood transfusion, the patient eventually survived AFE. PMD was confirmed after pathological examination of the placenta.

**Conclusions:**

While FGR can be monitored by color Doppler, our case echoed previous reports that IUFD may be unpreventable even under intensive surveillance in PMD cases. Although AFE is usually considered unpredictable, PMD can result in cumulative risk factors contributing to AFE. Whether a specific link exists between the pathophysiology of PMD and AFE requires further investigation.

**Supplementary Information:**

The online version contains supplementary material available at 10.1186/s12884-022-05261-2.

## Background

Placental mesenchymal dysplasia (PMD) is a rare pathological finding that manifests as an enlarged placenta covered with grape-like vesicles, which needs to be differentiated from molar pregnancies. PMD usually presents with a normal fetus, but is associated with 52% preterm delivery, 33% fetal growth restriction (FGR), 17% preterm premature rupture of membranes [1], and 29% intrauterine fetal demise (IUFD) [2]. Although surveillance with color Doppler is reasonable in the management of FGR, whether intensive ultrasound monitoring can prevent IUFD is still under debate.

As for maternal implications, 12.8% of PMD cases are associated with hypertensive disorders including preeclampsia, HELLP syndrome and eclampsia [3], whereas amniotic fluid embolism (AFE) has never been described in association with PMD. AFE is characterized by the triad of sudden blood pressure drop with desaturation, profound coagulopathy, and correlation with the timing of delivery. Although AFE is regarded unpredictable and unpreventable, it is notable that PMD can result in cumulative risk factors contributing to AFE. Herein, we report a case of PMD, in which the fetus eventually demised in utero, and the mother subsequently experienced AFE. To our knowledge, this is the first reported case of PMD complicated by AFE.

## Case presentation

A 43-year-old woman with no systemic disease, gravida 4, para 1, conceived after the transfer of three frozen embryos. By the 8th week of gestation, a single gestational sac along with a thickened placenta was recorded. At 14th week gestation, the placenta thickened to 3.48 cm and presented with multiple vesicles. The fetus was nevertheless grossly normal. The initial differential diagnosis included partial mole, complete mole with a co-twin, Beckwith-Wiedemann syndrome (BWS), and PMD. Afterwards, blood test revealed an extremely high maternal serum human chorionic gonadotropin (HCG) level of 192,817.9 mIU/mL (MoM: 6.51) and an elevated alpha-fetoprotein (AFP) level of 397.2 ng/mL (MoM: 9.57). Amniocentesis confirmed a normal karyotype of 46, XX. Methylation-specific polymerase chain reaction (MS-PCR) showed no abnormal methylation in the imprinting control region *H19* and *KvDMR* of chromosome 11p15, indicating that the fetus was not a case of BWS. Therefore, PMD was highly suspected.

**Fig. 1:**
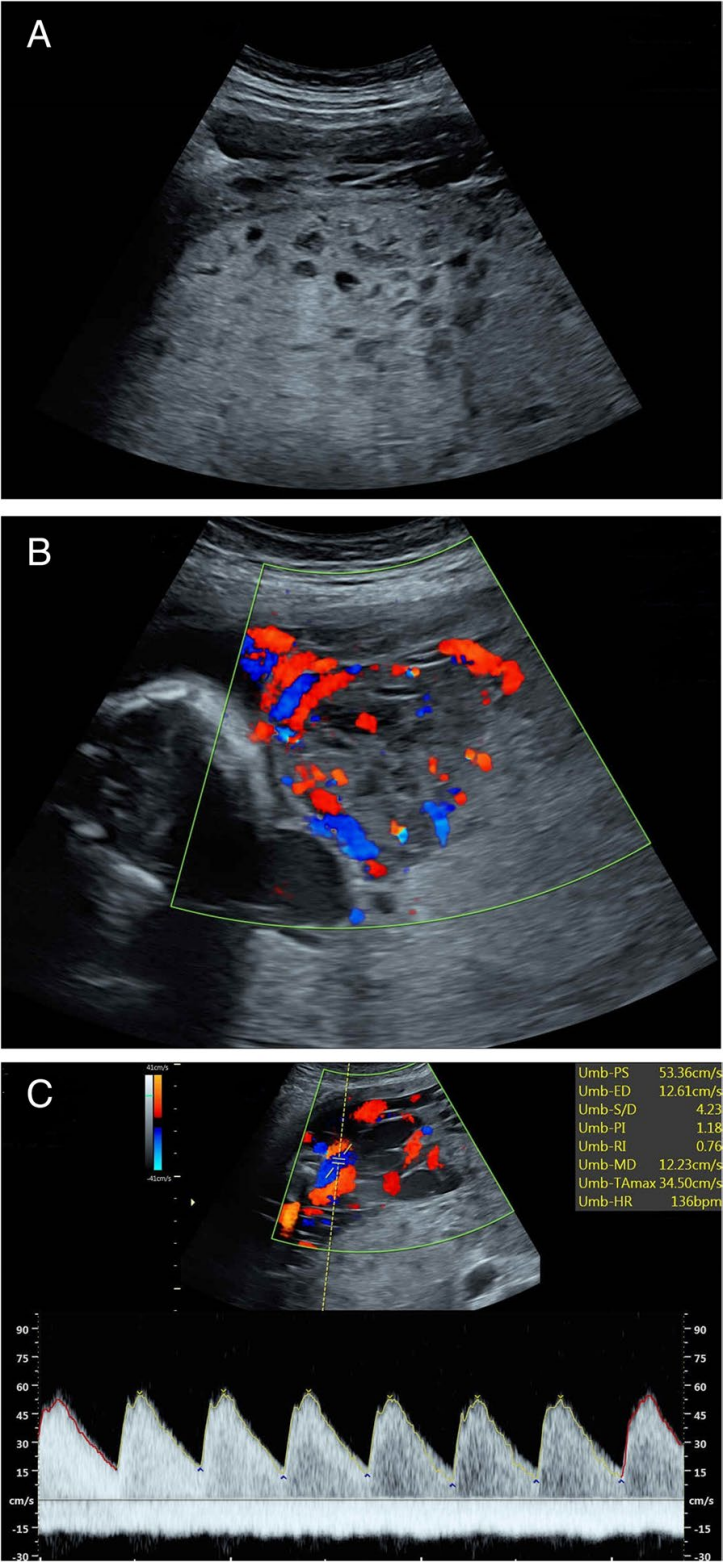
**A** Diffused sono-lucent vesicles in the enlarged hydropic placenta. **B** Scattered flow within the cysts, demonstrating a “stained glass sign”. **C** Aneurysmal dilated chorionic vessels compiling at the fetal surface with prominent blood flow.

Ultrasound surveillance was performed on a weekly basis. During the following weeks, we observed progressive enlargement of the hydropic placenta, and tortuous chorionic vessels with turbulent blood flow (Fig. 1 A, B, C). The hydropic placenta occupied the entire lower segment of the uterine cavity, resulting in placenta previa totalis. A symmetric growth delay of 1 week was first documented in the 21st week, while the amniotic fluid amount remained ample. The fetal umbilical artery and middle cerebral artery blood flow remained unremarkable until 25 weeks and 4 days gestation, when the patient came to our emergency department due to decreased fetal movements. After admission, ultrasound revealed placenta previa totalis, oligohydramnios and symmetric growth lag of 2 weeks. Color Doppler showed an unprecedented cerebroplacental perfusion ratio (CPR) of < 1, whereas the ductus venosus (DV) a-wave was normal. An elevated blood pressure and spot urine protein-creatinine ratio of 7.912 confirmed the diagnosis of preeclampsia. The ratio of soluble fms-like tyrosine kinase-1 to placental growth factor (sFlt-1/PlGF) was 124.6. Tococardiography showed a category 2 fetal heart rate tracing. Preparation for preterm delivery using magnesium sulfate and betamethasone was initiated. However, on the second day of admission, the fetus demised in utero.

After confirmation of fetal demise, the patient experienced uterine contractions along with antepartum hemorrhage. Emergent cesarean section was immediately performed under general anesthesia due to active vaginal bleeding of 228 g in an hour and insufficient fasting time. During the operation, within 3 min following placental expulsion, the patient experienced a sudden blood pressure drop (from 141/76 mmHg to 42/32 mmHg), bradycardia (heart rate dropped from 92 bpm to 59 bpm), followed by desaturation (SpO2 80–83%). Intraoperative transesophageal echocardiography revealed a dysfunctional right ventricle. Therefore, AFE was suspected. Inotropic agents, massive blood transfusion and coagulation factor VII were instantly administered. Meanwhile, her blood test revealed a low platelet count of 58,000/μL (reference range 150,000-400,000/μL), prolonged prothrombin time of 15.6 s (reference range 9.5–11.7 s), activated partial thromboplastin time (APTT) of 68.0 s (reference range 24.3–3.27 s), a low plasma fibrinogen level of 53.7 mg/dL (reference range 200–400 mg/dL), and an elevated fibrin degradation product (FDP) level of 369.3 μg/ml (reference range < 5 μg/ml). Rotational thromboelastometry was used for optimal correction of the coagulation profile. A Bakri intrauterine balloon was inserted, yet fresh blood oozing from the vagina persisted despite adequate uterine contractions. We discussed with the patient’s husband and the necessity to proceed with hysterectomy if the bleeding persisted. However, concerning the patient’s desire for fertility and her relatively acceptable vital signs following blood transfusion (BP: 100/62 mmHg, HR: 71 bpm), we decided to try trans-arterial embolization (TAE) firstly. The patient later underwent TAE, and her condition was finally stabilized. The estimated intraoperative blood loss was 890 ml, and postpartum blood loss was 1500 ml initially. The total blood products transfused included 14 units of pRBC, 18 units of fresh frozen plasma, 2 units of single donor platelets, and 50 units of cryoprecipitate. A tranexamic acid pump was administered with continuous infusion for 1 day. The patient was discharged on day 9 of hospitalization.

**Fig. 2:**
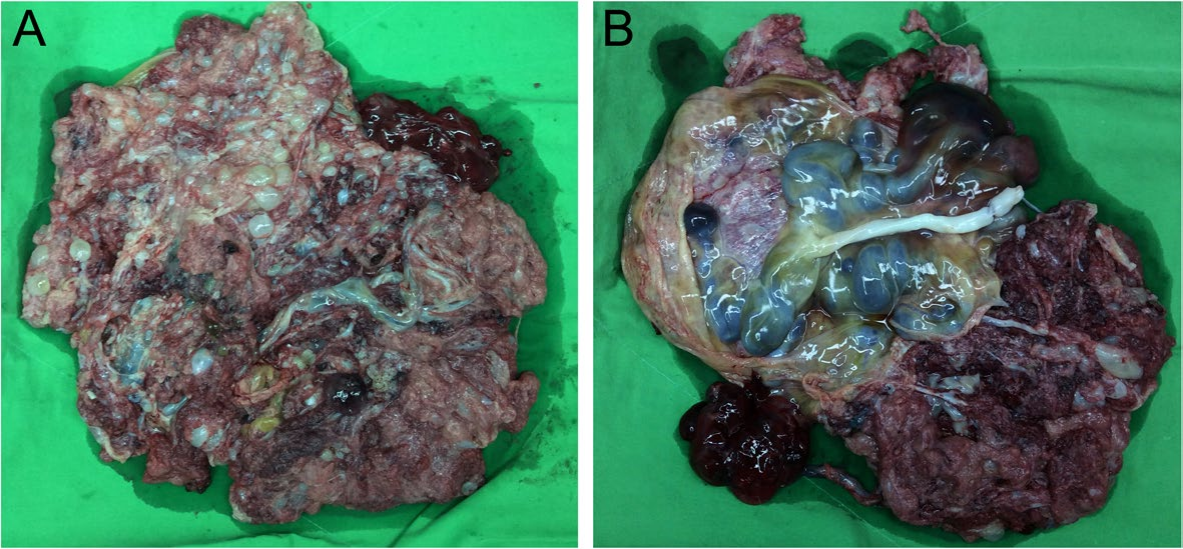
**A** The enlarged placenta was covered with multiple vesicles. **B** Marked dilatation of the chorionic vessels on the placental fetal surface. A chorioangioma was attached to the placenta with a feeding vessel.

**Fig. 3:**
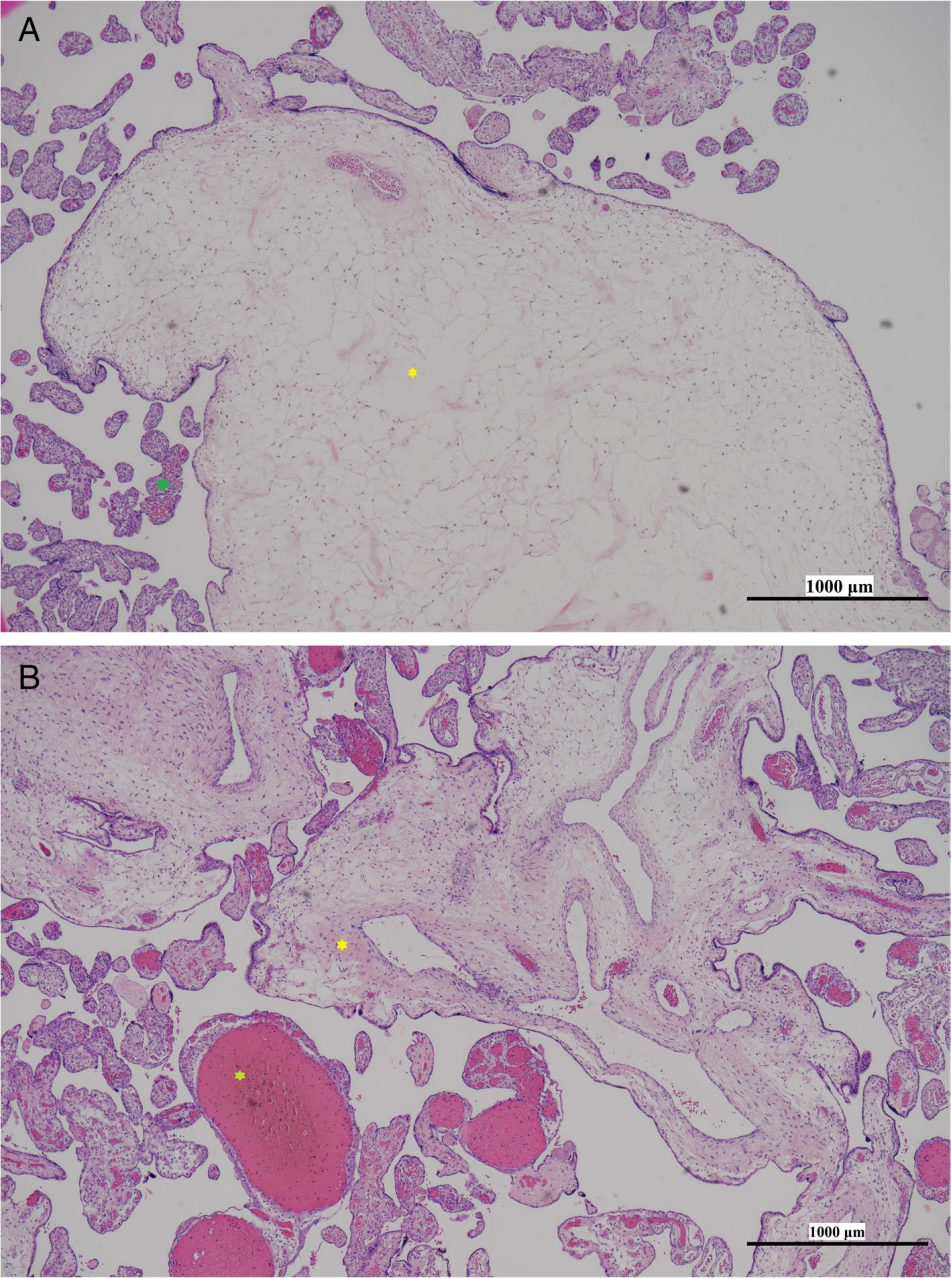
The microscopic images were acquired with a Nikon Eclipse Ni-U microscope equipped with a Nikon DS-Ri2 camera and NIS-Elements software; 40x; scale bars: 1000 μm. **A** Immunohistochemical staining showed hydropic villi filled with cisterns (yellow asterisk) (40x). By contrast, the distal villi (green asterisk) appeared normal, absent of trophoblastic proliferation (40x). **B** The chorionic plate vessels are dilated with thrombus (green asterisk) (40x), and thickened vessel walls are present with fibromuscular hyperplasia (yellow asterisk) (40x).

The demised fetus was grossly normal, yet the specific cause of death could not be assessed due to the patient’s refusal for autopsy. The placenta weighed 1400 g (MoM = 5.19), sized 31 × 21 × 8 cm^3^. Under gross inspection, the placenta appeared edematous and was composed of grape-like vesicles (Fig. 2 A). The fetal surface showed dilated chorionic vessels, up to 3 cm in diameter. Another 7 × 6 x 2cm^3^ chorioangioma was attached to the placenta via a feeding vessel (Fig. 2 B). Microscopically, the hydropic stem villi were abnormally enlarged, with central cistern formation. The terminal villi appeared normal, with no excessive proliferation (Fig. 3A, B). All the above findings were consistent with PMD. The specimen collected from the placenta was further examined by MS-PCR, with hypermethylation found on the *H19* gene and hypomethylation on the *KvDMR* gene, suggesting that androgenetic/biparental mosaicism is the etiology of PMD in this case.

## Discussion

PMD is a rare pathological finding of the placenta with an estimated incidence of 0.002–0.02% [4, 5]. It can be detected as early as 8–9 weeks’ gestation, and early differentiation from gestational trophoblastic disease is crucial. Under color Doppler, the aneurysmal dilated chorionic vessels may demonstrate the “stained glass sign” [6], whereas in molar pregnancy, these areas tend to be absent of flow. Approximately 70% of patients with PMD present with abnormally elevated AFP levels and 38% with elevated HCG levels [1]. In contrast, patients with molar pregnancies typically have elevated HCG and normal AFP levels. Invasive diagnostic procedures, including chorionic villus sampling and amniocentesis, can provide information on fetal karyotype, while MS-PCR can further detect epigenetic changes [7]. The etiology of PMD includes congenital malformations of the mesoderm, molecular disruption of the imprinting genes of chromosome 11p15.5 associated with BWS, fetal aneuploidy, and androgenetic/biparental mosaicism [1, 8]. Among the reported cases of PMD, androgenetic/biparental mosaicism is the most common etiology, which happened to be the same etiology as in our case, as confirmed by MS-PCR [9].

According to the current literature, the diagnosis of PMD is mainly based on histological findings. A key feature that distinguishes PMD from molar pregnancy is that trophoblastic proliferation is typically absent in PMD, whereas trophoblastic proliferation with myometrial invasion is commonly observed in molar pregnancies [1]. Our patient presented with dilated, edematous stem villi with central cisterns and thickened fibromuscular stem vessels, whereas the terminal villi were normal. Our findings are consistent with the characteristics of PMD.

PMD is associated with 52% preterm delivery, 33% FGR, and 17% preterm premature rupture of membrane [1]. Ishikawa et al. reported a 29% rate of IUFD at a median gestational age of 31 weeks [2]. The cause of fetal death is likely to be multifactorial. Pham et al. proposed that diffused vascular malformations may lead to different extents of shunting and obstructive thrombosis within the vessels, resulting in poor perfusion and chronic hypoxia of the fetus [10]. Rupture of the inappropriately dilated chorionic vessels or major thrombotic events occurring within the umbilical cord may also cause sudden IUFD [10]. In addition, an increased expression of the vascular endothelial growth factor-D (*VEGF-D*) gene on chromosome Xp22.31 may be a consequence of chronic fetal hypoxia [4, 10]. The VEGF overexpression may contribute to the hypervascularity and chorangiosis commonly seen in PMD placentas. This explains the formation of a large chorioangioma attached to the hydropic placenta, as observed in our case [10].

Whether intensive monitoring can prevent fetal death in patients with PMD is still under investigation. Given the high risk of FGR in PMD cases, it is reasonable to incorporate color Doppler imaging into the management of PMD. However, 56% of IUFD cases occur in the absence of FGR [10]. In our patient, a CPR ratio < 1 was first documented 1 day ahead of IUFD, and the aortic isthmus and DV a-wave remained normal throughout the course. Therefore, we speculated that in addition to chronic hypoxia, a thromboembolic event was more likely the cause of fetal demise in our case. These results reflected the unpredictability of IUFD in patients with PMD.

Regarding maternal implications, 12.8% of PMD cases are associated with maternal hypertensive disorders, including preeclampsia, HELLP syndrome, and eclampsia [3]. Our patient presented with unprecedented episodes of hypertension and proteinuria along with an elevated sFlt-1/PlGF ratio of 124.6 at 25 weeks’ gestation. The imbalanced maternal serum anti-angiogenic markers could be a consequence of the chronic hypoxic fetus and placenta, eventually leading to preeclampsia [11, 12]. Given that early onset preeclampsia is typically associated with placental insufficiency and trophoblastic invasion defects, the surveillance of preeclampsia should be performed in patients with PMD.

AFE is a clinical diagnosis based on the triad of a sudden blood pressure drop with desaturation, profound coagulopathy, and correlation with the timing of delivery after excluding other potential causes. In our case, severe pulmonary embolism causing desaturation and right ventricular strain, leading to cardiogenic shock, should be ruled out in the first place. However, such extent of pulmonary embolism would also result in severe shunting (V/Q mismatch), causing profound desaturation despite ventilation support and should not have recovered in a short period of time. Our patient experienced an episode of desaturation (SpO2 81%) under general anesthesia with mechanical ventilation. Her desaturation recovered within 5 min following the administration of norepinephrine 25 mcg in two doses followed by hydrocortisone 100 mg and oxygen support. This clinical course can be better explained by sudden pulmonary vasoconstriction triggered by the anaphylactoid reaction from the exposure to amniotic materials, rather than cardiogenic shock resulting from large pulmonary emboli. Second, as the patient had experienced frequent uterine contractions followed by vaginal bleeding, given that she had placenta previa totalis, placental abrution should be considered in this scenario. Nevertheless, we did not find the presence of retroplacental hematoma after placenta expulsion, nor were there any signs of Couvelaire uterus intraoperatively. Finally, hemorrhage-associated disseminated intravascular coagulation (DIC) should also be excluded in this case. However, patient’s hypotension and desaturation were not precipitated by tachycardia. The total blood loss was 228 g antepartum and 890 ml during the surgery. Given that the patient had also received 4 units of packed-RBC transfusion intraoperatively, this amount of blood loss was less likely to be responsible for the desaturation and profound DIC observed in our patient. After the exclusion of potential causes, AFE was strongly suspected, as our case fulfilled the diagnostic criteria proposed by the Society for Maternal-Fetal Medicine and the Amniotic Fluid Embolism Foundation, namely: 1) desaturation (peripheral SpO2 < 90%) and hypotension (SBP < 90 mmHg); 2) clinical onset within 30 min of delivery of the placenta; 3) afebrile during labor; and 4), documentation of overt DIC with a score ≥ 3 using the Modified International Society on Thrombosis and Hemostasis scoring system for overt DIC in pregnancy [13].

Most AFE risk factors are associated with conditions that increase disruption of the maternal-fetal interface, including cesarean section, instrumental vaginal delivery, placental anomalies (previa, accreta and abruption), polyhydramnios, cervical laceration and uterine rupture [14, 15]. Other risk factors are advanced maternal age, preeclampsia or eclampsia, multiple gestations, and fetal distress [14, 15]. First, our patient presented with an enlarged placenta that occupied the entire lower segment of the uterus, resulting in placenta previa totalis, leaving cesarean section as the only option for delivery. Second, as the bulky placenta was widely attached to the endometrium, an increased extent of maternal-fetal interface disruption may also occur during manual expulsion of the placenta, thereby increasing the risk of AFE. In hindsight, an “en caul” delivery could have possibly avoided the breach of the maternal-fetal interface. Furthermore, in addition to the advanced maternal age of our patient, imbalanced maternal serum angiogenic markers resulted from the chronically hypoxic placenta may also lead to preeclampsia, another known AFE risk factor [14]. Finally, whether certain antigenic substances that are more prone to trigger an anaphylactoid reaction in the maternal serum exist in the PMD placenta remains a question. Given that no specific material causing AFE has been identified in the current literature, our hypothesis cannot be verified.

To the best of our knowledge, this is the first reported case of PMD complicated with IUFD and AFE. While FGR can be monitored by color Doppler, our case echoed previous reports that IUFD may be unpreventable even under intensive surveillance in PMD cases. Finally, although AFE is considered unpredictable, it is noteworthy that PMD could result in cumulative risk factors that contribute to AFE. Whether a specific link exists between the pathophysiology of PMD and AFE requires further investigation.

## Supplementary Information

**Additional file 1:** **Supplementary Fig. 1.** Ultrasound confirmed placenta previa totalis. The image of placenta previa totalis confirmed by ultrasound on the day of admission at 25 weeks and 4 days gestation.

**Additional file 2:** **Supplementary Fig. 2.** Treatment course. The timeline of the treatment course after amniotic fluid embolism.

## Data Availability

The data and materials are available from the corresponding author under reasonable request.

## Abbreviations

PMD: Placenta mesenchymal dysplasia
AFEA: mniotic fluid embolism
AFP: Alpha fetoprotein
BWS: Beckwith-Wiedemann Syndrome
CPR: Cerebroplacental perfusion ratio.
DV: Ductus venosus
FGR: Fetal growth restriction
HCG: Human chorionic gonadotropin
IUFD: Intrauterine fetal demise
MS-PCR: Methylation-specific polymerase chain reaction
SFlt-1/PlGF: soluble fms-like tyrosine kinase-1 to placental growth factor ratio
VEGF: Vascular endothelial growth factor

## References

[1] Nayeri UA, West AB, Grossetta Nardini HK (2013). Systematic review of sonographic findings of placental mesenchymal dysplasia and subsequent pregnancy outcome. Ultrasound ObstetGynecol.

[2] Ishikawa S, MorikawaM YT (2015). Prospective risk of stillbirth in women with placental mesenchymal dysplasia. J Obstet Gynaecol Res.

[3] Kodera C, Aoki S, Ohba T, Higashimoto K, Mikami Y, Fukunaga M, Soejima H, Katabuchi H (2021). Clinical manifestations of placental mesenchymal dysplasia in Japan: a multicenter case series. J Obstet Gynaecol Res.

[4] Arizawa M, Nakayama M (2002). Suspected involvement of the X chromosome in placental mesenchymal dysplasia. Congenit Anom (Kyoto).

[5] Zeng X, Chen MF, Bureau YA, Brown R (2012). Placental mesenchymal dysplasia and an estimation of the population incidence. Acta Obstet Gynecol Scand.

[6] Kuwata T, Takahashi H, Matsubara S (2014). 'Stained-glass' sign for placental mesenchymal dysplasia. Ultrasound Obstet Gynecol.

[7] Chen CP, Su YN, Lin MH (2014). Detection of altered methylation status at 11p15.5 and 7q32 in placental mesenchymal dysplasia. Taiwan J Obstet Gynecol.

[8] Kaiser-Rogers KA, McFadden DE, Livasy CA (2006). Androgenetic/biparental mosaicism causes placental mesenchymal dysplasia. J Med Genet.

[9] Colpaert RM, Ramseyer AM, Luu T, Quick CM, Frye LT, Magann EF (2019). Diagnosis and Management of Placental Mesenchymal Disease. A review of the literature. Obstet Gynecol Surv.

[10] Pham T, Steele J, Stayboldt C (2006). Placental mesenchymal dysplasia is associated with high rates of intrauterine growth restriction and fetal demise: a report of 11 new cases and a review of the literature. Am J Clin Pathol.

[11] Herraiz I, Simón E, Toldos Ó (2017). Angiogenesis-related biomarkers (sFlt-1/PlGF) in placental mesenchymal dysplasia. J Matern Fetal Neonatal Med.

[12] Levine RJ, Maynard SE, Qian C (2004). Circulating angiogenic factors and the risk of preeclampsia. N Engl J Med.

[13] Clark SL, Romero R, Dildy GA, Callaghan WM, Smiley RM, Bracey AW (2016). Proposed diagnostic criteria for the case definition of amniotic fluid embolism in research studies. Am J Obstet Gynecol.

[14] Kramer MS, Rouleau J, Baskett TF, Joseph KS, Maternal Health Study Group of the Canadian Perinatal Surveillance System (2006). Amniotic-fluid embolism and medical induction of labour: a retrospective, population-based cohort study. Lancet.

[15] Abenhaim HA, Azoulay L, Kramer MS, Leduc L (2008). Incidence and risk factors of amniotic fluid embolisms: a population-based study on 3 million births in the United States. Am J Obstet Gynecol.

