# Supplementary figures and images for: Placental mesenchymal dysplasia complicated with sudden fetal demise and amniotic fluid embolism: a case report
Source: BMC Pregnancy Childbirth. 2022 Dec 9;22:927. doi: 10.1186/s12884-022-05261-2 (PMC9733268; doi:10.1186/s12884-022-05261-2)

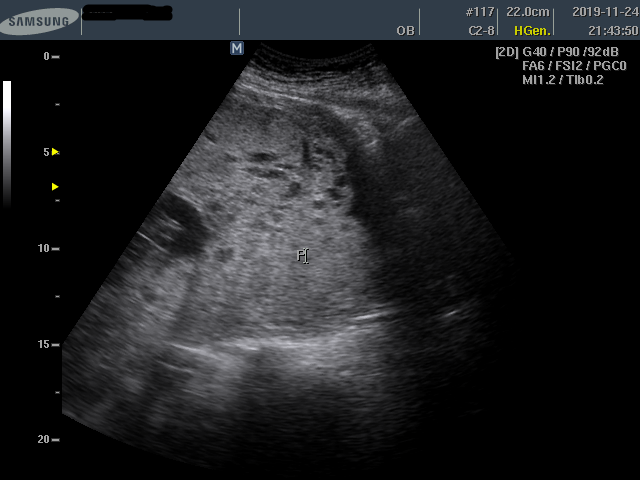

Supplement: Supplementary file 1 — Additional file 1: Supplementary Fig. 1. Ultrasound confirmed placenta previa totalis. The image of placenta previa totalis confirmed by ultrasound on the day of admission at 25 weeks and 4 days gestation. [file 12884_2022_5261_MOESM1_ESM.tif]

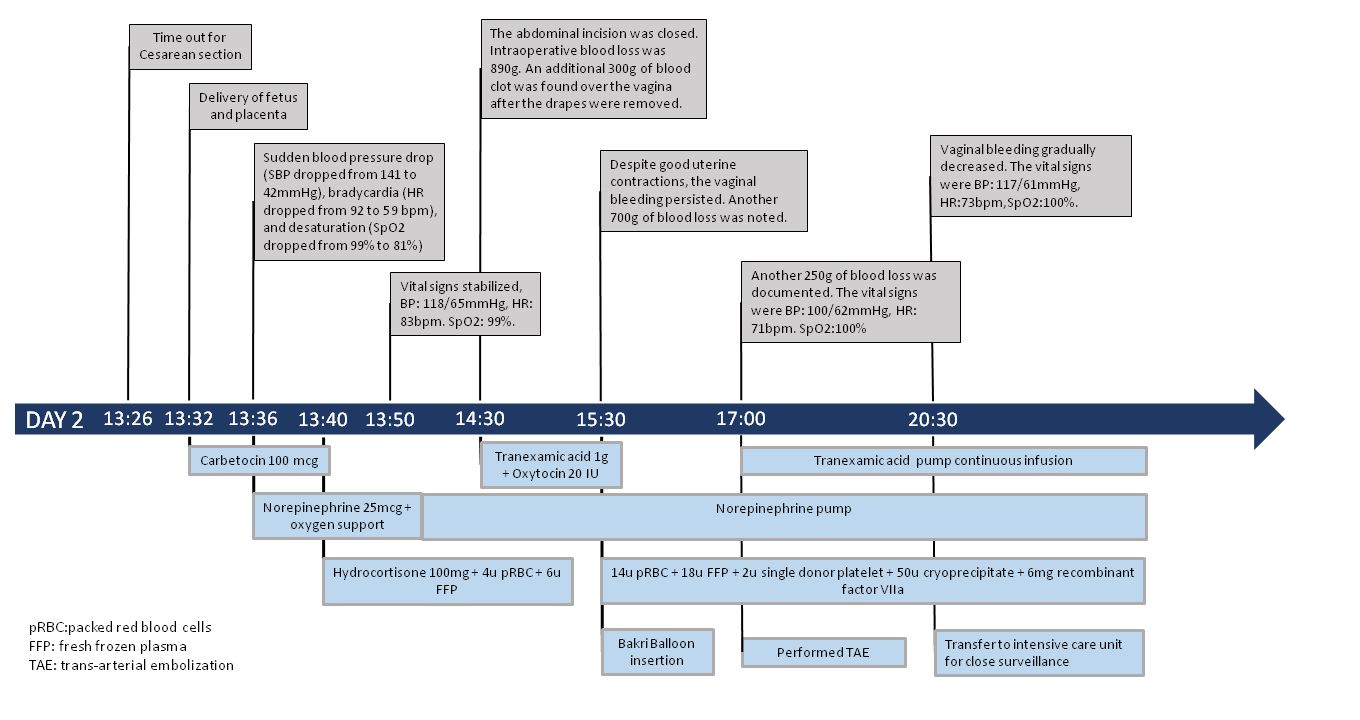

Supplement: Supplementary file 2 — Additional file 2: Supplementary Fig. 2. Treatment course. The timeline of the treatment course after amniotic fluid embolism. [file 12884_2022_5261_MOESM2_ESM.tif]
